# Towards Drug Repurposing in Cancer Cachexia: Potential Targets and Candidates

**DOI:** 10.3390/ph14111084

**Published:** 2021-10-26

**Authors:** Joana M. O. Santos, Alexandra C. Costa, Tânia R. Dias, Setareh Satari, Maria Paula Costa e Silva, Rui M. Gil da Costa, Rui Medeiros

**Affiliations:** 1 Molecular Oncology and Viral Pathology Group, Research Center of IPO Porto (CI-IPOP)/RISE@CI-IPOP (Health Research Network), Portuguese Oncology Institute of Porto (IPO Porto)/Porto Comprehensive Cancer Center (Porto.CCC), 4200-072 Porto, Portugal; joana.oliveira.santos@ipoporto.min-saude.pt (J.M.O.S.); alexandra.costa@ipoporto.min-saude.pt (A.C.C.); tania.dias@ipoporto.min-saude.pt (T.R.D.); setareh.satari@ipoporto.min-saude.pt (S.S.); rmcosta@fe.up.pt (R.M.G.d.C.); 2 Faculty of Medicine, University of Porto (FMUP), 4200-319 Porto, Portugal; mpaula.silva@ipoporto.min-saude.pt; 3 Research Department of the Portuguese League against Cancer–Regional Nucleus of the North (Liga Portuguesa Contra o Cancro–Núcleo Regional do Norte), 4200-177 Porto, Portugal; 4 Palliative Care Service, Portuguese Oncology Institute of Porto (IPO Porto), 4200-072 Porto, Portugal; 5 Postgraduate Programme in Adult Health (PPGSAD), Department of Morphology, Federal University of Maranhão (UFMA), São Luís 65080-805, Brazil; 6 LEPABE-Laboratory for Process Engineering, Environment, Biotechnology and Energy, Faculty of Engineering, University of Porto, Rua Dr. Roberto Frias, 4200-465 Porto, Portugal; 7 Centre for the Research and Technology of Agro-Environmental and Biological Sciences (CITAB), Inov4Agro, University of Trás-os-Montes e Alto Douro (UTAD), Quinta de Prados, 5000-801 Vila Real, Portugal; 8 Virology Service, Portuguese Oncology Institute of Porto (IPO Porto), 4200-072 Porto, Portugal; 9 Biomedical Research Center (CEBIMED), Faculty of Health Sciences, Fernando Pessoa University, 4249-004 Porto, Portugal

**Keywords:** cancer, cachexia, drug repurposing, drug repositioning

## Abstract

As a multifactorial and multiorgan syndrome, cancer cachexia is associated with decreased tolerance to antitumor treatments and increased morbidity and mortality rates. The current approaches for the treatment of this syndrome are not always effective and well established. Drug repurposing or repositioning consists of the investigation of pharmacological components that are already available or in clinical trials for certain diseases and explores if they can be used for new indications. Its advantages comparing to de novo drugs development are the reduced amount of time spent and costs. In this paper, we selected drugs already available or in clinical trials for non-cachexia indications and that are related to the pathways and molecular components involved in the different phenotypes of cancer cachexia syndrome. Thus, we introduce known drugs as possible candidates for drug repurposing in the treatment of cancer-induced cachexia.

## 1. Introduction

Cachexia is a syndrome that involves different tissues and metabolic pathways, and it is related with poor prognosis in cancer patients. Anorexia, asthenia, sarcopenia, and anaemia are present features in cancer cachexia along with a reduction of response to anabolic signals, domination of a catabolic state, energy expenditure imbalance, and systemic inflammation. Continued loss of weight, adipose tissue, and skeletal muscle are the results of these systemic actions [1].

According to the international consensus published in 2011, cancer cachexia is defined by ≥5% weight loss in the previous 6 months or weight loss ≥ 2% with either a body mass index (BMI) of 20 kg/m^2^ or sarcopenia. Moreover, cancer cachexia has three phases, including pre-cachexia, cachexia, and refractory cachexia [2].

The presence of a variety of divergent pro-cachectic mechanisms makes it complex to define a single standard treatment for cachectic patients [2]. According to the latest American Society of Clinical Oncology (ASCO) guideline on the management of cancer cachexia, treatment interventions can be divided into three groups: nutritional interventions, pharmacological interventions, and other interventions, such as exercise [3]. As stated in ASCO guidelines of management of cancer cachexia, there are no FDA-approved drugs to ameliorate the complications of cancer cachexia, and clinicians may choose not to prescribe any medications for the treatment of cancer cachexia. However, there are some evidence-based recommended drugs, such as megestrol acetate, which improves appetite and weight gain, although it is mainly correlated to increased adipose tissue mass [3].

Drug repurposing or repositioning is the strategy to use drugs approved or in clinical trials for certain diseases and to investigate if they can be used for new indications [4]. The advantages of drug repurposing comparing to the new development of drugs are the considerable amount of time and cost reduction. In addition, there is already available information concerning the safety of the drugs [4]. As an example of successful cases of drug repurposing, there is thalidomide, which was initially used for morning sickness during pregnancy but eventually withdrawn from the market because of its teratogenicity and is now repurposed for refractory multiple myeloma excluding pregnant women [4].

In this work, based on the different phenotypes and molecular components associated with cancer cachexia, we retrieved drugs from drug-target databases that can potentially modulate those pathways in order to achieve more treatment options for cancer-induced cachexia.

## 2. Materials and Methods

### 2.1. Selection of Phenotypes and Pathways/Molecular Components Involved in Cachexia Syndrome

In order to retrieve the cachexia’s phenotypes and the molecular pathways and components involved in each one, we searched for review papers on PubMed regarding the pathophysiology of cancer cachexia. From the search performed [1,5,6,7], we retrieved the phenotypes and respective components presented in Table 1.

### 2.2. Selection of Pharmacological Candidates

In order to select pharmacological candidates that can potentially be useful in the treatment of cachexia, we made a search based on Swanson’s ABC model. This model says that if A is connected with B, and B is related with C, then C may have a novel connection with A [8]. In order words, if cachexia is connected with a certain target, and that target is related to a certain drug, then that drug may have a novel connection with cachexia.

Based on this model, we searched for drugs that interact with the targets/pathways involved in each cachexia’s phenotype using drug-target interaction databases, namely IUPHAR/BPS Guide to Pharmacology and Drugbank [9,10,11]. For the drugs obtained from the search on these databases, we retrieved only the drugs that are approved or in clinical trials for non-cachexia indications. For that, we also used Clinicaltrials.gov. When additional information for each drug was necessary, the data were extracted from PubMed literature. Additionally, we also searched on PubMed if there were pre-clinical studies for the drugs obtained in cancer cachexia. All these searches were performed from March 2021 to April 2021.

## 3. Results

### 3.1. Inflammation

Systemic inflammation plays a central role in the genesis of cachexia. Pro-inflammatory mediators can be released by tumor cells or by host immune cells in response to the presence of the tumor and thus activate pathways that lead to muscle and adipose tissue wasting, altered energy balance, and dysregulation of the homeostatic control in the central nervous system [5,6]. Among the pro-inflammatory mediators, the ones mainly implicated in the pathogenesis of cachexia are TNF-α, IL-6, IL-1, IL-8, and IFN-γ [1,7].

Thus, since inflammation is a key factor in the development of cachexia, blocking the synthesis or action of pro-inflammatory mediators to treat or ameliorate cachexia in cancer patients has been attempted with mixed results [12]. From all the drugs obtained in the drug-target interaction databases regarding the inflammation process in cancer-related cachexia (Table 2), we will only describe in more detail the ones with reported side effects and are in more advance stages of clinical development and relevant information.

#### 3.1.1. TNF-α

Adalimumab

This is an anti-TNF-α monoclonal antibody approved by Food and Drug Administration (FDA) and European Medicines Agency (EMA) for the treatment of rheumatoid arthritis, juvenile idiopathic arthritis, psoriatic arthritis, ankylosing spondylitis, Crohn’s disease, plaque psoriasis, and hidradenitis suppurativa [13]. Its rare side effects include worsening or initiation of congestive heart failure, lupus-like syndrome, lymphoma, cytopenias, worsening or initiation of multiple sclerosis/neurological diseases, pancytopenia, and increased liver transaminases [14]. In a retrospective study performed in patients with psoriasis, the administration of adalimumab significantly increased the weight and BMI compared to the control group [15].

Certolizumab Pegol

This is an anti-TNF-α monoclonal antibody approved by FDA and EMA for the treatment of rheumatoid arthritis, Crohn’s disease, axial spondyloarthritis, and psoriasis [16]. As side effects, there is a risk of infection and production of autoantibodies [17]. Leucopaenia, pancytopaenia, thrombocytopaenia, seizure disorder, neuritis, and peripheral neuropathy are rare [17]. It is also contraindicated in cases of heart failure, Parkinson’s disease, or demyelinating conditions [17].

Chloroquine

This is an antimalarial drug that also inhibits TNF-α. Chloroquine is also approved for the treatment of rheumatoid arthritis and systemic lupus erythematosus [18]. The side effects, such as reduced visual acuity, diplopia, bilateral loss of vision, paranoia, hallucinations, suicidal ideations, pruritus, and photosensitivity, are usually associated with high doses [18].

Inamrinone (Amrinone)

This is a phosphodiesterase inhibitor with the capacity to inhibit TNF-α, already approved by FDA to treat congestive heart failure. Side effects include thrombocytopenia, gastrointestinal and cardiovascular effects, hepatic toxicity, and hypersensitivity [19]. In a study using septic rats, it was shown that the ones treated with daily injections of amrinone for 5 days prevented the inhibition of protein synthesis in muscle induced by sepsis [20]. However, another study performed in rats showed that amrinone did not alter cyclic adenosine monophosphate (cAMP) levels and rates of overall proteolysis in soleus and extensor digitorum longus muscles [21]. 

Pomalidomide

This is an analogue of thalidomide that also has the capacity to inhibit the production of TNF-α and IL-6 by monocytes [22]. It is an approved drug by FDA and EMA for multiple myeloma and Kaposi sarcoma [22,23]. The most common side effects are neutropenia, thrombocytopenia, anemia, and fatigue [24]. Venous thromboembolisms are also reported, with an incidence similar to other immunomodulatory drugs [24]. Infections and primarily pneumonia are also observed [24].

Glycyrrhizic Acid

This is a natural product derived from the root of *Glycyrrhiza glabra* and an antagonist of TNF-α [25]. It is approved by FDA and used for the treatment of premenstrual syndrome, viral infections, anti-lipidemic, antihyperglycemic, peptic ulcer, and other stomach diseases [26]. Some of the side effects reported when consumed in high doses include hypermineralocorticoidism with sodium retention and potassium loss, edema, increased blood pressure, cardiac complaints, and depression of the reninangiotensin-aldosterone system [26]. A study showed that in tumor-bearing mice, the treatment with glycyrrhizin alone or combined with cisplatin reversed the loss of body weight [27].

#### 3.1.2. IL-6

Sirukumab

This is an anti-IL6 monoclonal antibody and a phase 3 clinical candidate for rheumatoid arthritis, polymyalgia rheumatica, and temporal arteritis and also phase 2 clinical candidate for major depressive disorder [28,29]. The associated side effects include nasopharyngitis, elevated liver enzymes, injection site erythema, and upper respiratory tract infections [30].

#### 3.1.3. IL-1

Canakinumab

This is a monoclonal antibody that binds to IL-1β. It is approved by familial cold autoinflammatory syndrome 1, juvenile idiopathic arthritis—systemic, and Muckle–Wells syndrome [31]. The most common side effects involve headache, vertigo, diarrhea, nausea, musculoskeletal pain, rhinitis, nasopharyngitis, and bronchitis.

Rilonacept

This is a fusion protein consisting of the binding domains of the IL-1 receptor and the IL-1 receptor accessory protein [32]. It is approved by FDA for Muckle–Wells syndrome, familial cold autoinflammatory syndrome 1, and CINCA syndrome [33]. The more common side effects reported include bleeding, body aches or pain, cough, and fever, among others.

Anakinra

This is a recombinant, non-glycosylated human interleukin-1 receptor antagonist approved by FDA and EMA for rheumatoid arthritis [32]. The most common side effects are reaction at the injection site, worsening of rheumatoid arthritis, upper respiratory tract infection, headache, nausea, diarrhea, sinusitis, arthralgia, flu like-symptoms, and abdominal pain. A study showed that Anakinra, when administrated in colon-26 adenocarcinoma-bearing Balb/c-mice, reduced loss of body weight [34].

### 3.2. Skeletal and Cardiac Muscle Wasting

During cachexia, both skeletal and cardiac muscle wasting are observed, and their underlying mechanisms are intertwined [1,35]. The impaired protein synthesis and increased proteolysis that are observed in skeletal myoblasts and cardiomyocytes occurs via common pathways, such as autophagy, IGF-1 pathway, nuclear factor-kappa B (NF-kB), and up-regulation of UPS [1,35]. Therefore, the drugs that will be proposed in the next sections can be considered as potential candidates to ameliorate skeletal and cardiac muscle wasting. From all the drugs obtained in the drug-target interaction databases regarding the skeletal and cardiac muscle-wasting process in cancer-related cachexia (Table 3), we will only describe in more detail the ones with reported side effects and are in more advance stages of clinical development and relevant information.

#### 3.2.1. Autophagy

Skeletal muscle is one of the most metabolically active tissues in the body and is essential for a variety of different biological activities, such as movement, support to soft tissue, and respiration. A balance between protein synthesis and degradation is present in normal physiological states; however, it is commonly disrupted during the tumor progression. Indeed, extensive loss of skeletal muscle represents a key manifestation of cancer-associated cachexia. Cachexia primarily results from an acceleration of protein degradation, often combined with reduced protein synthesis in skeletal muscle [36].

Autophagy is one of the main promoters of proteolysis in skeletal muscle and plays an important role in cancer cachexia [1], involving an extremely refined collection of altered organelles, abnormal protein aggregates, and pathogens, similar to a selective recycling centre [37]. This process is regulated by AMP-activated protein kinase (AMPK), which maintains energy homeostasis through the regulation of cellular metabolism. AMPK is able to promote autophagy, under glucose starvation conditions, by activating an autophagy-initiating kinase, unc-51 like autophagy activating kinase 1 (ULK1) involved in autophagosome formation. Additionally, it was demonstrated that an inactivation of ULK1 resulted in a decrease of autophagy in muscle cells. Therefore, we think ULK1 is a potential target in autophagy, as it interacts with different molecules in the process of autophagy. 

However, using autophagy inhibitors in cancer patients is still a controversial issue since, on one hand, autophagy can suppress malignant transformation by decreasing the production of reactive oxygen species and DNA damage, and on the other hand, autophagy can support proliferation, tumorigenicity of cancer stem cells, and increase drug resistance [38]. Thus, due to its dual-role in cancer, it should be well-thought-out before giving an autophagy inhibitor in a cancer cachectic patient.

ULK1 Inhibitors

Fedratinib

Federatinib (TG 101348) is an FDA-approved drug for intermediate-2 and high-risk primary and secondary myelofibrosis. The most common side effects include: anaemia, thrombocytopenia, neutropenia, nausea, diarrhea, constipation, bleeding, urinary tract infection, headache, muscle spasms, fatigue or asthenia [39,40].

Critzotinib

Critzotinib is an FDA-approved type-1 kinase inhibitor, originally approved for treatment of anaplastic lymphoma kinase-positive non-small cell lung carcinomas. The most common adverse effects include: vomiting, diarrhea, nausea, constipation, abdominal pain, elevated transaminases, rash, oedema, and fatigue [41].

Fostamatinib

Fostamatinib is an FDA drug approved for the treatment of chronic immune thrombocytopenia. It has also completed phase 3 clinical trials for rheumatoid arthritis and phase 2 for a range of solid tumors [42]. Dizziness, diarrhea, nausea, frequent bowel movement, hypertension, and increased liver enzymes are amongst the very common adverse effects of the drug [43].

#### 3.2.2. Ubiquitin-Mediated Proteasome Degradation System (UPS)

UPS is one of the main mechanisms of protein degradation during muscle wasting [1]. This system comprises several components, such as ubiquitin activating enzymes (E1), ubiquitin-conjugating or carrier enzymes (E2), ubiquitin ligases (E3), deubiquitinating enzymes (DUBs), and the 26S proteasome that comprises two subcomplexes, such as 20S core protein and 19S regulatory particle [44]. Due to the key role of this system in muscle wasting, its components may provide pharmacological targets.

Bortezomib

Bortezomib has the capacity to inactivate the catalytic site on β subunits, which form the active 20S core [45]. It is approved by FDA and EMA for multiple myeloma. Side effects include nerve problems, nausea, fever, low blood cell counts, and liver problems, among others. Some clinical efficacy has been detected in patients with systemic lupus erythematosus, and other autoimmune disorders [46,47]. A study performed in rats showed that Bortezomib reduced NF-kB and proteasome activity in skeletal muscle but did not prevent weight loss, muscle wasting, and reduced food intake; however, 20S activation was not accessed [48].

Carfilzomib

It is a proteasome inhibitor approved by FDA and EMA to treat multiple myeloma. The most common adverse reactions reported are fatigue, anemia, nausea, thrombocytopenia, dyspnea, diarrhea, and pyrexia [49]. A study showed that carfilzomib in combination with z-VAD-fmk in a mouse model of cancer-induced cachexia reduced muscle wasting, tumor burden, modulated metabolism, increased glucose, albumin, and total proteins levels and lowered triglyceride fatty acids levels. It also induced more spontaneous physical activity and longer survival when compared to the control group. Gastrocnemius muscle had reduced proteolysis and apoptosis [50].

Ixazomib

This drug inhibits 20S proteasome activity, and it is approved by FDA and EMA to multiple myeloma systemic light-chain amyloidosis [51]. Some of the side effects include hepatic damage, fetal harm in pregnant women, diarrhea, thrombocytopenia, and skin/subcutaneous disorders [52]. For multiple myeloma, it is in phase 3, and for bladder cancer and renal cell carcinoma, it is in phase 1/2. It can cause several hepatic diseases and also fetal harm. In a mouse model for Duchenne muscular dystrophy, it was shown that Ixazomib reduced inflammation in muscles and increased the number of fibres [53]. The expression of dystrophin and utrophin was increased, and the expression of osteopontin and transforming growth factor beta (TGF-β) decreased [53].

#### 3.2.3. Calcium-Activated Protease Calpains

The muscle wasting observed in cachexia is associated with several proteolytic pathways and processes, including the calpain system [54]. These proteins are associated with the initiation of protein breakdown during cachexia since calpain-dependent cleavage of myofilaments is considered the initial step in muscle proteolysis [54,55]. Calpains comprise a family of calcium-activated cysteine proteases, which cleave at exposed regions between domains of proteins affecting muscle in synergisms with UPP [55,56]. Therefore, inhibitors of calpains might also protect skeletal muscle from cachexia-induced apoptosis [55]. The drugs associated with this phenotype are mentioned in Table 3.

#### 3.2.4. Insulin Resistance

Insulin resistance is one of the risk factors involved in the development of cancer and is also seen in initial stages of cachexia, resulting in muscle wasting [1]. Insulin receptor dephosphorylation is performed by protein tyrosine phosphatases (PTPases). Various studies have reported that the insulin resistance in type 2 diabetes and obesity, both in animal models and humans, is accompanied with an increase in PTPases activity and increases in the level of expression of defined members of the PTP family, especially protein tyrosine phosphatase 1B (PTP1B). Therefore, an inhibitor to PTP1B might act as a potential treatment for insulin resistance [57,58]. The drugs associated with this phenotype are mentioned in Table 3.

#### 3.2.5. PIF

PIF is a glycoprotein that was initially isolated from the murine cachexia-inducing MAC16 tumor model, but it was also found to be present in the urine of patients with diverse range of carcinomas (pancreas, breast, ovary, lung, rectum, and liver) who suffer from cachexia [59]. PIF is involved in many biological functions, from controlling protein catabolism in cancer cachexia to regulating hepatic gene expression [60]. Despite that, PIF’s normal role is at embryonic development [61]. It is thought that PIF’s production is ceased before birth but that certain tumors regain the ability to synthesize it through glycosyl transferases that restrict expression during embryogenesis [62].

There are several therapeutic approaches that can be considered to target alterations promoted by PIF in cancer-associated cachexia such as inhibit the release and nuclear translocation of NF-κB or its binding to the DNA in the nucleus. In addition, downregulation of phospholipase A2 (PLA2) or lipoxygenases or even inhibit protein kinase C (PKC) are considered possible approaches [62].

NF-kB Antagonists

PIF is capable of initiating muscle protein degradation as a result of up-regulation of the ATP-ubiquitin-dependent proteolytic pathway [63]. Mechanistically, PIF is able to decrease cytosolic IκBα, NF-kB inhibitor protein. This leads to an increased NF-κB migration to the nucleus and consequent activation of forkhead box O (FOXO), which results in an augment of transcription of ubiquitin ligase genes (*FBXO32* and *TRIM63*) that consequently promote muscle protein degradation [64].

Lestaurtinib

Lestaurtinib (CEP-701) is an orally available tyrosinase kinase inhibitor that is in clinical trials for the treatment of psoriasis and a variety of neoplastic disorders (including leukemia and breast cancer) [65,66]. Moreover, CEP-701 is also a potent NF-kB blocker via the inhibition of IκBα phosphorylation [67]. The main reported side effects, at least in one study with myelofibrosis patients, were anaemia and thrombocytopenia as well as nausea, vomiting, and diarrhea [68].

Parthenolide

Parthenolide is a sesquiterpene lactone present in the flowers and leaves of the plant feverfew (*Tanacetum parthenium* L.). Parthenolide has been used in clinical trials for the diagnostic of Allergic Contact Dermatitis [69]. This drug can inhibit the activation and release of NF-κB and prevent its binding to the DNA [70]. Additionally, dimethylaminoparthenolide (DMAPT), which is a water-soluble and orally bioavailable analogue of parthenolide, has shown to ameliorate wasting syndrome in HPV16-transgenic mice and in a transgenic mammary tumor model [71,72].

Acetylsalicylic Acid

Acetylsalicylic acid (ASA), also known as aspirin, is an FDA-approved drug to treat pain and reduce fever and inflammation [73]. Moreover, it is also used as a preventive treatment for heart attacks, strokes, and chest pain (angina). ASA has also been associated with NF-κB inhibiton [74]. Some of the side effects include upset stomach, drowsiness, and mild headache.

Sulfasalazine

Sulfasalazine is an FDA-approved drug for the treatment of inflammatory bowel diseases [75]. It acts as a potent and specific inhibitor of NF-kB. Sulfasalazine has proven to interfere with IkBα phosphorylation, which suggests that it has a direct effect either on IkBα or on an upstream signal [76]. Adverse effects include gastrointestinal effects, dizziness, headache, and rash; myelosuppression can also occur [77]. 

Phospholipase A2 Antagonists

Protein metabolism induced by PIF leads to the release of arachidonic acid (AA) from membrane phospholipids. It is thought that this mechanism involves PLA2 [78]. Consequently, the release of AA serves as a signal to activate PKC family of serine/threonine kinases that act as intracellular signals of PIF action on the proteasome [79].

Anagrelide

Anagrelide is an FDA-approved drug for the treatment of thrombocythaemia (elevated levels of platelets) in patients with myeloproliferative neoplasms [80]. It can inhibit the release of AA from phospholipases through PLA2 inhibition [81]. So far, anagrelide has been associated with some cases of interstitial pneumonitis [82].

Budesonide

Budesonide is an FDA-approved drug, since 1994, for the treatment of inflammatory conditions of the lungs and intestines (such as asthma, COPD, Chron’s disease, and ulcerative colitis). It is a glucocorticoid that inhibits PLA2 by decreasing AA formation and inhibiting NF-κB [83]. Side effects may include headache, indigestion, back pain, and cold symptoms.

Hydrocortisone

Hydrocortisone, also known as cortisol, is an FDA- and EMA-approved drug to treat immune, allergic, and neoplastic disorders. Like the other glucocorticoids mentioned above, hydrocortisone is a PLA2 antagonist [83]. There is evidence that low-dose hydrocortisone infusion attenuates the systemic inflammatory response in human septic shock [84]. The most frequent adverse effects include atrophy, striae, rosacea, perioral dermatitis, acne, and purpura [85].

PKC Antagonists

PKC is another crucial molecule to PIF-induced expression of the ubiquitin-proteasome pathway [86]. PKC activation can arise from the conversion of 15-lipoxygenase (15-LOX) to 15-hydroxyeicosatetraenoic acid (15-HETE), which is an important intracellular signal for the induction of the ubiquitin-proteasome proteolytic pathway [87].

Bryostatin 1

Bryostatin 1 is a potent modulator of PKC activity that is on clinical trials for Alzheimer’s disease and different types of cancer. Prolonged exposure of tumor cells to bryostatin-1 promotes PKC inhibition through ubiquitin-mediated proteasomal degradation from the cell [88]. The major side effects associated are nausea, myalgias, and vomiting [89].

Tamoxifen

Tamoxifen is an FDA-approved drug, since 1977, for the treatment of oestrogen receptor positive breast cancers [90]. Furthermore, tamoxifen is also able to regulate PKC through several mechanisms, although it is not able to interact with the active site of the enzyme [91]. Most reported side effects include hot flushes, joint pains, headaches, and vaginal dryness [92].

### 3.3. Adipose Tissue Depletion

From all the drugs obtained in the drug-target interaction databases regarding the adipose tissue-depletion process in cancer-related cachexia (Table 4), we will only describe in more detail the ones with reported side effects and that are in more advanced stages of clinical development and relevant information.

#### 3.3.1. Lipolysis

Although muscle wasting is the main manifestation of cachexia that impacts quality of life, the loss of adipose tissue is also a feature of cancer cachexia, which contributes to the negative imbalance [93]. Lipolysis, which is the breakdown of adipose tissue, is possibly the most evident mechanism of adipose tissue that contributes to cancer cachexia [94]. The enhancement of lipolysis in cachectic cancer patients is driven by an overactivation of lipases, such as adipocyte triglyceride lipase (ATGL) and hormone-sensitive lipase (HSL). ATGL is responsible for the initial steps in triglyceride breakdown, forming diacylglyceride (DAG) and free fatty acids (FFA), while HSL finalizes the hydrolysis producing FFAs and glycerol [93,94]. Additionally, it would also be important to stabilize adipose tissue metabolism to preserve skeletal muscle mass since infiltration of adipose tissue into skeletal muscle can also contribute to wasting of this tissue [6].

ABX-1431

ABX-1431 was described as a selective inhibitor of monoacylglycerol lipase, which is a serine hydrolase that plays a crucial role catalysing the hydrolysis of monoglycerides into glycerol and fatty acids [95]. Despite some adverse effects, such as headache, somnolence, and fatigue, this drug was evaluated in human clinical trials to improve the treatment of some diseases, like Tourette syndrome or chronic motor tic disorder, functional dyspepsia, post herpetic neuralgia, diabetic peripheral neuropathy, small fiber neuropathy, and post-traumatic neuralgia [95,96,97]. Moreover, ABX-1431 was demonstrated to be crucial in the control of lipid metabolism through the inhibition of the monoacylglycerol lipase in neurologic disorders [96,97].

#### 3.3.2. Inhibition of Lipogenesis

Reduced lipogenesis contributes for adipose tissue depletion in cachectic patients [98]. Lipogenesis consists in the de-novo fatty acid synthesis [98]. This process starts from high levels of glucose in circulation that stimulate the release of insulin from the pancreas [98]. Then, insulin promotes the uptake of glucose by adipocytes, stimulates glycolytic and lipogenic enzymes, and stimulates the expression of important genes for lipogenesis [98]. Glucose metabolization provides Acetyl-CoA which is the substrate for fatty acids synthesis [98]. Finally, fatty acids are esterified to a glycerol molecule and form triglycerides that, in white adipose tissue (WAT), will be stored as energy reserve [98,99]. Additionally, WAT can also import fatty acids using the lipoprotein lipase, which will catabolize circulating lipoproteins into fatty acids. Decreased activity of lipoprotein lipase has been observed in cancer cachexia [99].

#### 3.3.3. WAT Browning

While lipolysis represents a depletion of adipose tissue principally in WAT mass, brown adipose tissue (BAT) is a site of heat production (thermogenesis) contributing to cancer cachexia by increasing energy expenditure [6,94]. Furthermore, in cachectic condition, there is an increase on the expression of the thermogenic marker UCP1 in BAT [93]. In cachexia, the proton electrochemical gradient that leads to ATP synthesis is disrupted by the activation of UCPs proteins, leading to heat production and energetic inefficiency [6].

WAT browning occurs in response to β-adrenergic stimulation and also in response to chronic peroxisome proliferator-activated receptor gamma (PPARγ) agonist stimulation [100]. PPARγ activation enhances UCP1 expression driving to BAT formation and regulating the thermogenic activity [101,102]. On the other hand, PPARγ antagonists inhibit the browning process and also decreas the expression of thermogenic key markers [103].

β-adrenergic Blockers

β_3_-adrenergic receptor plays a crucial role in lipolysis and thermogenesis regulation, being the principal signalling pathway that active WAT browning [104,105]. WAT tissue normally has low levels of UCP1 expression; however, WAT displays thermogenic capacity with high levels of UCP1 expression upon certain signals; this process is called “browning” [105]. Therefore, β-adrenergic blockers can reduce the severity of cachexia by decreased lipolysis and WAT browning [104,105]. Then, there exist some β_3_-adrenoceptor antagonists approved by the FDA that are used in other diseases but could be potential drugs in the treatment of cancer cachexia. On the other hand, β-adrenergic receptor-blocking drugs can be associated with some adverse effects, and the more common are bronchospasm, heart failure, prolonged hypoglycaemia, bradycardia, heart block, and intermittent claudication; neurological reactions include depression, fatigue, and nightmares [106].

Bupranolol

Bupranolol is a β_3_-adrenoceptor antagonists approved by the FDA and is mainly used in hypertension and tachycardia treatment [107,108,109]. In an experimental study, it was demonstrated that bupranolol significantly reduced lipolysis since levels of glycerol and non-esterified free fatty acids were reduced [108]. As an β-adrenergic receptor-blocking drug, the side effects associated are bronchospasm, heart failure, prolonged hypoglycaemia, bradycardia, heart block, and intermittent claudication; neurological reactions including depression, fatigue, and nightmares [106].

Levobunolol

Levobunolol is a β_3_-adrenoceptor antagonist approved by the FDA and used in intraocular pressure, chronic open-angle glaucoma, and ocular hypertension [110]. The adverse effects more frequently associated with this drug are fatigue, dizziness, heart palpitations, and bradycardia [111].

Nadolol

Nadolol, a β_3_-adrenoceptor antagonist approved by the FDA, is also used in diverse treatments, such as those for angina pectoris, infantile hemangioma, and hypertension [112,113,114]. Cutaneous vascular lesion, bradycardia, hypotension, and hypoglycemia are the more common adverse events correlated with this drug [115].

PPARγ Antagonist

Diclofenac

Diclofenac is a PPARγ antagonists that is already approved by the FDA [116]. PPARγ antagonists could be important to improve the cachexia treatment since they inhibit the browning process and also decrease the expression of thermogenic key markers [103]. The adverse symptoms are mostly skin reactions, loss of taste, and joint pain/swollenness [116].

### 3.4. Liver

In the initial stages of tumor development (or pathogen infection/local tissue injury), the organism responds by initiating an acute phase response (APR) [117]. However, “too much of a good thing is a bad thing”; therefore, a prolonged or severe APR can lead to detrimental effects [118]. During the APR, there is a reprioritization of hepatic protein synthesis, which results in an augment of positive acute phase proteins (p.e. serum amyloid A, fibrinogen, and C reactive protein) accompanied by a decrease in plasma concentrations of negative acute phase proteins (such as albumin and transferrin) [119]. What is interesting is that, when placed on nutritional support, malnourished patients have an accelerated synthesis of positive acute phase protein, which in turn contributes to the loss of lean tissue [120].

APR is induced by pro-inflammatory cytokines (such as IL-6, IL-1, TNF, and IFN-γ) produced by innate immune cells in response to the systemic inflammation process. These cytokines elicit the activation of two major signalling pathways: JAK/STAT3 and mitogen-activated protein kinase (MAPK) pathway, which consequently stimulate the liver to produce acute-phase proteins [121].

Since systemic inflammation is the process that triggers these alterations in the liver, one of the therapeutic approaches to take in this case is to include an anti-inflammatory supplementation in programs of nutritional support. These drugs will target the signalling pathways involved, mainly IL-6 and IL-1, that are the crucial inflammatory mediators. They were addressed before the section on inflammation.

### 3.5. Altered Energy Balance

Cachexia is characterized as an energy balance disorder, in which is verified a decreased in energy intake and/or an increase of energy expenditure [7]. This syndrome is driven by a combination of metabolic changes, such as inflammation, excess catabolism, and elevated energy expenditure and a decreased of food intake [6]. Hypothalamic exposure to the various inflammatory stimuli leads to an alteration in the neuronal population activity that control metabolic processes, such as proteolysis and lipolysis, and regulate appetite leading to weight loss, anorexia, and skeletal muscle atrophy [5]. Importantly, the brain is crucially involved in the altered energy balance in cancer patients since its mediators are significantly involved in the control of food intake through regulation of appetite, satiation, taste, and smell of food and, consequently, are partially responsible for the anorexia of the cancer patient [5,6]. Several studies of central nervous system regulation in cancer cachexia focus in the administration of neuromodulator peptides, such as ghrelin [5]. Another crucial component in the regulation of appetite and metabolism is the neuropeptide calcitonin gene-related peptide (CGRP) that has been shown to be involved with the decrease of food consumption [122]. From all the drugs obtained in the drug-target interaction databases regarding the altered energy balance process in cancer-related cachexia (Table 5), we will only describe in more detail the ones with reported side effects and that are in more advanced stages of clinical development and relevant information.

#### 3.5.1. Ghrelin Agonists

Ghrelin was identified as the endogenous ligand of the growth hormone secretagogue (GHS) receptor; it is able to increase muscle mass through the GH/insulin-like growth factor-1 (IGF-1) axis and promote adiposity, and it is also a potent stimulator of food intake. Therefore, studies demonstrated that administration of ghrelin agonists have numerous positive effects, including increased appetite, body weight, and muscle strength and improved fatigue, gastrointestinal functions, and hypoglycaemia [123]. For these reasons, ghrelin has been suggested as a treatment to prevent cachexia [124].

Pralmorelin (GHRP-2)

An example of one ghrelin agonist is the drug pralmorelin (GHRP-2), which is already approved in Japan and increases growth hormone release from the pituitary [123]. This drug seems to be a promising agent for the treatment of severe anorexia nervosa as a chronic condition [123]. Moreover, it was demonstrated that the GHRP-2 administration in arthritic rats decreased the serum IL-6 levels; then, this drug apparently also had anti-inflammatory effects in arthritic rats [124]. Besides, in another study, it was shown that GH-releasing peptides improve cardiac dysfunction and cachexia and suppress stress-related hormones and cardiomyocyte apoptosis in rats with heart failure [125]. There are no common reported side effects [126].

Macimorelin

Macimorelin is also a ghrelin agonist approved by the FDA used to improve adult growth hormone (GH) deficiency and is associated with multiple side effects: headache, nausea, vomiting, diarrhea, abdominal pain, dyspepsia, nasopharyngitis, and pain in extremity [127].

Ibutamoren (MK-0677)

Ibutamoren (MK-0677), studied in a clinical trial concerning the treatment of fibromyalgia, is another oral ghrelin receptor agonist that demonstrated ability to maintain normal GH secretion and increased lean body mass in normal subjects [128]. An in-vivo study demonstrated an increase of food intake and body weight through the activation of the hypothalamic mRNA expression of NPY and agouti-related protein (AgRP) and a decrease of the UCP1 levels in brown adipose tissue, leading to a consequent decrease of energy expenditure [129]. The most frequent side effects reported include an increase in appetite, mild lower extremity edema, and muscle pain [130].

Ulimorelin (TZP-101)

Ulimorelin (TZP-101) is also a ghrelin receptor agonist conducted in diverse clinical trials associated to gastrointestinal motility disorders [131,132]. This drug displayed a promising pharmacokinetic, pharmacodynamic, and safety profile, and the adverse effects associated are headache, lower abdominal pain, diarrhea, and dizziness [131].

#### 3.5.2. Inhibitor of Monocarboxylate Transporter 1 (MCT1)

AZD3965

Lactate inhibits the secretory function of ghrelin-producing gastric cells as a regulator of energy intake and is important to reduce lactate activity in cachexia [133,134]. AZD3965 is a potent inhibitor of MCT1, which is a lactate transporter. This drug was submitted to clinical trials in order to improve diffuse large B-cell lymphoma and Burkitt lymphoma, demonstrating a potent effect on lactate transport inhibition [133,135]. The most commonly reported side effects were nausea and fatigue [136].

#### 3.5.3. Calcitonin Gene-Related Peptide (CGRP) Receptor Antagonist

CGRP is a neuropeptide that has been shown to be involved with the decrease of food consumption and altered calorimetric parameters and plasma metabolic hormone levels, thus confirming that CGRP plays a pivotal role in the regulation of appetite and metabolism [122]. Therefore, inhibition of CGRP neurons protects against loss of lean body, which may explain the influence of these neurons in cachexia development and their effect on food intake [137].

Rimegepant

Rimegepant, an FDA-approved drug, is a CGRP receptor antagonist used in the treatment of migraines. Nausea, dizziness, urinary tract infection, and liver injury are the most commonly reported adverse events in patients treated with Rimegepant [138]. As a CGRP blocker, Rimegepant has good efficacy and safety [138].

Ubrogepant

Ubrogepant is another FDA-approved drug with the potential to inhibit CGRP receptors; thus, it was described as a potential antagonist of CGRP receptors that is effective and safe for the treatment of acute migraine [139]. GRP promote the dilation of the cerebral arteries and mediate neurogenic inflammation of the dura; as an CGRP receptor antagonist, ubrogepant mainly acts on the smooth muscle cells of the microvascular wall to control peripheral vascular resistance. The most common adverse effects were headache, oropharyngeal pain, nasopharyngitis, nausea, dizziness, diarrhea, and fatigue [139].

Atogepant

Atogepant is also a CGRP receptor antagonist used in diverse clinical trials, most of which are related to migraine treatment [140]. The adverse effects of these drugs are very similar to the side effects caused by the other CGRP receptor antagonists, which include nausea, dizziness, and vomiting [140,141].

### 3.6. Neuroinflammation

The maintenance of energy homeostasis is crucial for long-term survival [142]. The central melanocortin system is located principally in the arcuate nucleus (ARC) of the hypothalamus, which is an area of relative permeability of the blood–brain barrier, giving exposure to circulating indicators of disease activity, including inflammatory cytokines [143]. ARC has 2 subsets of neurons that have opposite effects on energy homeostasis. First, the anorexigenic proopiomelanocortin (POMC) neurons release α-melanocyte stimulating hormone (α-MSH) in synapses, which consequently binds to melanocortin receptors (MC4R), leading to a decrease in food-seeking behavior, an increase in basal metabolic rate, and a decrease in lean body mass. Second, the orexigenic NPY and AgRP neurons are natural inverse agonist of the MC4R, producing a decrease in the constant tone that POMC neurons place on restraining appetite [144,145].

Cytokines released during inflammation and malignancies act on the central nervous system to modulate the function of several key neurotransmitters (such as serotonin and leptin), leading to both altered appetite and metabolic rate [146]. When the systemic inflammation is sensed by the hypothalamus, it reacts by inducing sickness behavior and activating the nicotine anti-inflammatory system in order to restrict tumor growth by starvation or restore the immune surveillance. As it is well known, cancer cells possess the ability to surpass these mechanisms of inflammation resolution and promote neurochemical events that persistently activate POMC neurons, resulting in neuroinflammation [147].

Some potential drugs that target these specific mechanisms involved in neuroinflammation will be presented next, but it is important to have in mind that this imbalance in the neuronal network is mainly due to an exacerbated increase in systemic inflammation, so anti-inflammatory drugs, already addressed in a previous section, will also have beneficial effects. From all the drugs obtained in the drug-target interaction databases regarding the neuroinflammation process in cancer-related cachexia (Table 6), we will only describe in more detail the ones with reported side effects and that are in more advanced stages of clinical development and relevant information.

#### Serotonin Antagonists

One of the neurotransmitters that contributes to energy balance by triggering satiety is serotonin [148]. In anorexic and cachectic cancer patients, increased levels of tryptophan (serotonin precursor) were found in plasma and cerebrospinal fluid [149]. These anorectic effects of serotonin are mediated by the melanocortin system. The two serotonin receptors are located in the ARC; 5-HT2cR is expressed by POMC neurons, while on the contrary, 5-HT1bR is expressed by NPY neurons [150].

Pizotifen

Pizotifen acts as a 5-HT2cR antagonist. Despite not being approved by the FDA and EMA, it is available in several countries for the prophylactic treatment of migraines and cluster headaches [151]. Some of the side effects reported include drowsiness, tiredness, and weight gain [152].

Trazodone

Trazodone is an FDA-approved drug, since 1981, to treat major depressive disorders. It acts as a 5-HT2cR antagonist [153]. Other not officially approved uses of trazodone include treatment of bulimia, fibromyalgia, and degenerative diseases [154]. Furthermore, trazodone has the advantage of not decreasing sexual function or promoting insomnia, which are common features associated to this type of drug. Side effects like sedation, orthostatic hypotension, and headaches were reported [155].

Ziprazidone

Ziprazidone is a second-generation antipsychotic approved by the FDA to treat schizophrenia and related psychotic disorders. This compound acts as an antagonist of 5-HT2cR [156]. The most common adverse reactions include somnolence, respiratory tract infections, extrapyramidal symptoms, dizziness, akathisia, abnormal vision, asthenia, vomiting, headache, and nausea.

Clozapine

Clozapine is an antipsychotic agent approved by the FDA and used in the treatment of resistant schizophrenia. It is a serotonin antagonist with high-affinity to 5-HT2c receptor subtype [157]. Side effects include agranulocytosis, weight gain, diabetes, myocarditis, and seizures [158].

Olanzapine

Olanzapine is a second-generation anti-psychotic agent approved by the FDA to treat schizophrenia and other psychotic illnesses, like bipolar disorder. It targets 5-HT2cR and is very similar to clozapine [157,159]. Weight gain, hyperglycemia, and increased cholesterol and triglycerides are among the most common side effects reported.

### 3.7. Impaired Barrier Function and Malabsorption

Cachexia also promotes alterations in gastrointestinal function impairing gut-barrier function at several levels, including altered intestinal morphology, decreased renewal for various cell linages, depressed immunity, and increased gut permeability associated with decreased expression of tight junctions [160]. Moreover, gut-barrier function and also gut microbiota composition and function are significantly altered in cancer cachexia, leading to malabsorption [161]. Cancer cachexia is also associated with gastrointestinal mucosal atrophy, which leads to endotoxin absorption, poor wound healing, and sepsis [5]. Cachexia progression is correlated with barrier dysfunction and also with increased bacterial lipopolysaccharide levels [161]. Studies revealed that cachectic patients had a significantly higher rate of microbial translocation than non-cachectic patients and healthy controls [161]. Besides, gut-barrier alterations may reinforce systemic inflammation due to the translocation of pro-inflammatory bacterial compounds [160]. Increased levels of pro-inflammatory cytokines, such as TNF-α, IFN-γ, and diverse interleukins, have been demonstrated to increase paracellular permeability by impacting the expression or degradation of claudin and occludin tight-junction proteins [161]. The major macromolecules of tight junctions are occludins, claudins, and junction adhesion molecules [162]. Zonula occludens (ZO), ZO-1, is the main tight-junction protein that binds to the intracellular domain of occludins, playing a crucial role in sustaining the structure of tight junctions and consequently epithelial barrier function [163]. From all the drugs obtained in the drug-target interaction databases regarding the gastrointestinal tract process in cancer-related cachexia (Table 7), we will only describe in more detail the ones with reported side effects and that are in more advanced stages of clinical development and relevant information.

#### 3.7.1. Zonulin Inhibitor

Intestinal tight junctions are able to create gradients in order to promote an optimal absorption and transport of nutrients; besides, these molecules are crucial in the control of the paracellular antigens trafficking [164]. Tight junctions are dynamics structures that act in diverse key functions of the intestinal epithelium [164,165]. Zonulin was described as a main modulator of intracellular tight junctions. and it is known to reversibly regulate intestinal permeability by modulating intercellular tight junctions [164]. The expression of zonulin is augmented in some diseases, such as autoimmune conditions associated with tight junctions’ dysfunction, including celiac disease [164]. Therefore, zonulin can be used as a biomarker of impaired gut-barrier function and can be a potential therapeutic target [164,166].

Larazotide

Larazotide acetate (AT1001) is a synthetic eight amino acid peptide; it is orally administered and acts locally [165,166,167]. This drug is known to act as a tight-junction regulator acting as a zonulin inhibitor, and it is able to close leaky or open interepithelial junctions [165,167]. This drug is being studied in phase 3 clinical trials to evaluate the efficacy and safety of larazotide acetate for the relief of persistent symptoms in patients with celiac disease [165,167]. This drug seems to prevent opening of intestinal epithelial tight junctions induced by numerous stimuli, like cytokines, bacterial antigens, and gluten peptides [166]. Larazotide acetate was normally well tolerated, and it is not associated to serious adverse events, with headache and urinary tract infection among the most common [167].

#### 3.7.2. ZO-1 and Claudins Agonist

Tight junctions are constituted by three major groups of macromolecules, which are occludins, claudins, and junction-adhesion molecules [162]. Occludin and claudins interact with the ZO proteins, which are connected with the actin cytoskeleton, thus regulating cell-cycle control, and they are associated with cell polarity and permeability function [163,168]. ZO-1 is the major tight-junction protein that binds to the intracellular domain of occludins, and this interaction is crucial to maintain the structure of tight junctions and epithelial-barrier function [163]. Therefore, ZO-1 acts in both tight-junction and cell adhesion pathway being a key factor in the maintenance of the integrity of tight junctions’ complexes through linking claudins, occludins, and cytoskeletal proteins [163,168]. Moreover, several studies have indicated that TNF-α leads to the disruption of tight-junction assembly and decreases expression of ZO-1 [169].

Diacerein

Diacerein is an approved drug for the treatment of osteoarthritis although the use of diacerein is restricted due to the side effects, including severe diarrhea, dyspepsia, gastroesophageal reflux disease, and hemorrhoidal hemorrhage [170,171]. Diacerein also was under investigation in several clinical trials for the treatment of insulin resistance, diabetes mellitus (type 2), and diabetes-related complications [172]. Importantly, this drug it is metabolized to rhein, which reduces intestinal permeability by protecting intestinal epithelial tight-junction proteins ZO-1 and occludin, which alleviates the damage to the intestinal mucosa [162]. Besides, rhein enhances the expression of ZO-1 and occludin, repairs damaged tight junctions, and protects the intestinal barrier, which is important in cachectic patients [162,173].

Lubiprostone

Lubiprostone, an FDA-approved drug, is an activator of chloride channels-2 and a member of a class of compounds called prostones. It is a gastrointestinal-targeted bicyclic fatty acid that is able to enhance intestinal fluid secretion [174]. This drug is highly effective in treating constipation (chronic idiopathic constipation, irritable bowel syndrome with constipation), and the most common adverse events are diarrhea and nausea [175]. Furthermore, lubiprostone has been shown to have a positive effect on the intestinal-barrier function since oral administration of this drug significantly reduced the severity of colitis and reduced intestinal permeability [174]. It was demonstrated that lubiprostone increased the expression of claudin-1, which is crucial to the normal function of the epithelial barrier function. Moreover, this drug improved the IFNγ-induced decrease [174].

## 4. Discussion

**Figure 1:**
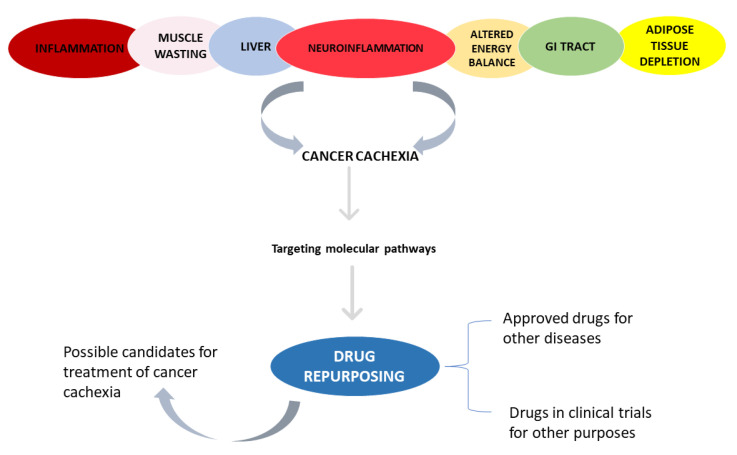
Schematic representation of the paper approach.

**Figure 2:**
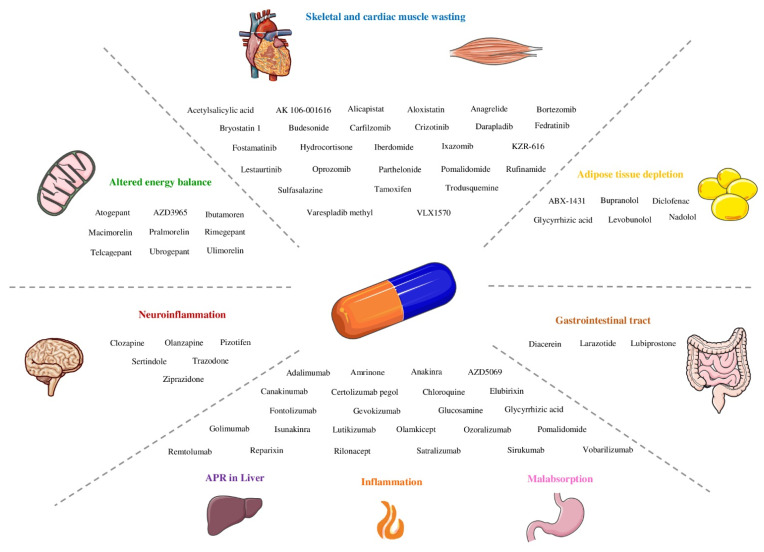
Drugs present in cachexia’s phenotypes.

Cachexia remains a major clinical challenge, with only few pharmacological options for the treatment of this syndrome, which are still far from being fully effective. Drug repurposing is a strategy that offers several advantages when comparing with the de-novo development of drugs. Taking this into consideration, this paper highlights drugs that have not been explored in clinical trials for cachexia and its related phenotypes (Figure 1) but targets key molecular components responsible for the cachexia syndrome (Figure 2) and that have been approved or in clinical trials for several other diseases. For that, we used IUPHAR/BPS Guide to Pharmacology and Drugbank to retrieve the drugs that act on the targets/pathways involved in each cachexia’s phenotype. These drug-target databases were selected since Drugbank incorporates data that are experimentally validated and approved as well as Guide to Pharmacology, which is subject to review and quality control [176,177,178,179].

Inflammation and muscle wasting are the pathways with more drugs that remain to be explored in the context of cachexia. For some of the phenotypes, such as acute phase response in liver, neuroinflammation, malabsorption, as well as impaired mitochondrial metabolism, the treatment with anti-inflammatory drugs it is also reported to be beneficial. Additionally, glycyrrhizic acid acts in both inflammation and adipose tissue wasting since it is an antagonist of TNF-α and induces lipoprotein lipase activity.Furthermore, pomalidomide acts on inflammation and muscle wasting due to is capacity to inhibit TNF-α, IL-6, and cereblon. We think that it should be of great interest to better explore those drugs in the context of cachexia since they intervene in more than one phenotype. For the phenotype related to low levels of circulating IGF-1, no drugs were found in the drug-target databases that could increase IGF-1 levels and that have not been tested in clinical trials related to cachexia. Regarding impaired mitochondrial metabolism, we were able to find some molecules involved in this phenotype, such as OXPHOS proteins, uncoupling proteins (UCP) 2/3, sarcoplasmic reticulum calcium ATPases (SERCA), and peroxisome proliferator-activated receptor-gamma coactivator (PGC)-1α, among others. Oxidative phosphorylation (OXPHOS) involves an electron transfer through the OXPHOS subunit proteins (complex III, IV, and V) generating an electrochemical gradient used to produce ATP [180]. In muscle wasting, decreased expression levels of OXPHOS subunit proteins leading to an impairment of mitochondrial activity has been observed [181,182]. Additionally, in skeletal muscle mitochondria, the uncoupling protein 2 and 3 (UCP) genes are overexpressed, promoting an inefficient ATP synthesis [183,184]. SERCA activity also increases promoting energy inefficiency since, during Ca^2+^ export to the cytosol, they consume the ATP associated with this process, generating Ca^2+^ overload [6]. Moreover, PGC-1α coactivates several transcription factors that regulate mitochondria biogenesis. It is reduced during cancer cachexia, resulting in loss of muscle mitochondrial content and ATP production [185]. 

However, for these molecular targets, we were not able to find any drugs that could be used for this purpose on the drug-target databases used in this paper. In the case of myostatin, all the drugs retrieved are in clinical trials for phenotypes related to cachexia.

From the drugs obtained in this study, adalimumab, amrinone, glycyrrhizic acid, anakinra, carfilzomib, ixazomib, parthelonide, pralmorelin, and ibutamoren already have pre-clinical studies in cancer cachexia; thus, we found these to be promising therapeutic approaches and should be further evaluated in clinical trials. Particularly with bortezomib, the pre-clinical study did not show any improvement in weight loss, muscle wasting, and reduced food intake, so we think that other pre-clinical studies should be performed.

We believe that the remaining drugs reported in this paper could be better explored in future drug-repurposing studies to understand if they are reliable to be administrated in cancer patients and effective at preventing or ameliorating cachexia. Some of these proposed drugs present some associated common side effects (e.g., diarrhea, vomit, among others). It is important to have in mind that the severity of the side effects varies among patients according to their disease stage, physical condition, and genetic background. However, for each patient, it should be assessed if drug benefits can overcome these side effects, and treatment should only be administered when this condition is verified.

Moreover, other strategies/drugs could be used for attenuation of the side effects, such as what happens with the use of megestrol acetate. When this drug is used in cancer cachectic patients, it has as side effects of diarrhea. Thus, to treat this side effect, it is recommended to eat low-fiber foods high in protein and calories or use drugs that can reduce or stop diarrhea. The same occurs with prednisolone and dexamethasone, which are prescribed to ameliorate anorexia symptoms and should be used simultaneously with proton pump inhibitors to avoid corticotherapy adverse effects. Additionally, new strategies on precision medicine/pharmacogenomics must be evaluated to identify patients that will benefit from those drugs and thus reduce the frequency of side effects.

Since incorporation of omics strategies may lead to significant advances on drug repurposing, future omics-based drug-repurposing studies should be performed for cancer cachexia in order to identify other relevant driver pathways and new drugs for cancer cachexia as well as to help inform decisions on efficacy and toxicity [186].

## 5. Conclusions

In this paper, we suggest possible drugs that could be potential candidates for future drug-repurposing studies. From the cachexia’s phenotypes presented in this paper, the ones with more drugs that could be further explored are inflammation and muscle wasting. Drugs that intervene in more than one phenotype, such as glycyrrhizic acid and pomalidomide, should be taken in consideration in order to improve the treatment of cancer cachexia. However, it should always be kept in mind that drug benefits should always overcome the side effects.

## Figures and Tables

**Table 1:** Cachexia’s phenotypes and respective molecular pathways and components.

Cachexia Phenotypes	Molecular Pathways and Components
Inflammation	Increased levels of tumor necrosis factor alpha (TNF-α), interleukin (IL)-6, IL-1, interferon gamma (IFN-γ), and IL-8
Skeletal and cardiac muscle wasting	Up-regulation of the ubiquitin-mediated proteasome degradation system (UPS)
	Autophagy
	Calcium-activated protease calpains
	Low circulating levels of insulin-like growth factor 1 (IGF-1)
	Insulin resistance
	Myostatin
	Proteolysis-inducing factor (PIF)
	Impaired mitochondrial metabolism
Adipose tissue depletion	Lipolysis
	Inhibition of lipogenesis
	Browning
Hepatic metabolic changes	Acute-phase response
Altered energy balance	Tumor metabolism and inflammation might increase resting energy expenditure and simultaneously decrease energy intake (anorexia), shifting the scale towards negative energy balance
Central neuroinflammation	Inflammatory cytokines bind to receptors on hypothalamic neuronal populations, triggering an acute illness response, leading to anorexia, weight loss, skeletal muscle-protein catabolism, and lipolysis. Neuropeptide Y (NPY), melanocortins, and serotonin involved.
Gastrointestinal tract malfunction	Impaired barrier function and malabsorption

**Table 2:** Molecular pathways and respective drugs involved in inflammation.

Phenotype	Molecular Pathways and Components	Drugs
Inflammation	TNF-α	Adalimumab
		Ozoralizumab
		Golimumab
		Certolizumab pegol
		Remtolumab
		Chloroquine
		Amrinone
		Pomalidomide
		Glycyrrhizic acid
	IL-6	Sirukumab
		Olamkicept
		Vobarilizumab
		Satralizumab
	IL-1	Lutikizumab
		Gevokizumab
		Canakinumab
		Rilonacept
		Isunakinra
		Anakinra
	IL-8	AZD5069
		Reparixin
		Elubirixin
	IFN-γ	Fontolizumab
		Glucosamine

**Table 3:** Molecular pathways and respective drugs involved in skeletal and cardiac muscle wasting.

Phenotype	Molecular Pathways and Components	Drugs
Skeletal and cardiac muscle wasting	UPS	Pomalidomide
		Iberdomide
		Bortezomib
		Carfilzomib
		Ixazomib
		Oprozomib
		VLX1570
		KZR-616
	Autophagy	Fedratinib
		Critzotinib
		Fostamatinib
	Calcium-activated protease calpains	Aloxistatin
		Alicapistat
	Insulin resistance	Trodusquemine
	PIF	Lestaurtinib
		Parthelonide
		Acetylsalicylic acid
		Sulfasalazine
		Anagrelide
		Varespladib methyl
		Darapladib
		AK 106-001616
		Budesonide
		Hydrocortisone
		Bryostatin 1
		Tamoxifen

**Table 4:** Molecular pathways and respective drugs involved in adipose tissue depletion.

Phenotype	Molecular Pathways and Components	Drugs
Adipose tissue depletion	Lipolysis	ABX-1431
	Inhibition of lipogenesis	Glycyrrhizic acid
	WAT browning	Brupanolol
		Levobunolol
		Nadolol
		Diclofenac

**Table 5:** Molecular pathways and respective drugs involved in altered energy balance.

Phenotype	Molecular Pathways and Components	Drugs
Altered energy balance	Ghrelin	Pralmorelin
		Macimorelin
		Ibutamoren
		Ulimorelin
	MCT1	AZD3965
	CGRP receptor	Rimegepant
		Ubrogepant
		Telcagepant
		Atogepant

**Table 6:** Molecular pathways and respective drugs involved in neuroinflammation.

Phenotype	Molecular Pathways and Components	Drugs
Neuroinflammation		Pizotifen
		Trazodone
	Serotonin	Ziprazidone
		Clozapine
		Olanzapine
		Sertindole

**Table 7:** Molecular pathways and respective drugs involved in altered gastrointestinal tract.

Phenotype	Molecular Pathways and Components	Drugs
Gastrointestinal tract: Impaired barrier function	Zonulin	Larazotide
	ZO-1 and claudins	Diacerein
		Lubiprostone

## Data Availability

Not applicable.
